# A Qualitative Study on Researchers’ Experiences after Publishing Scientific Reports on Major Incidents, Mass-Casualty Incidents, and Disasters

**DOI:** 10.1017/S1049023X21000911

**Published:** 2021-10

**Authors:** Johannes Nordsteien Svensøy, Helene Nilsson, Rune Rimstad

**Affiliations:** 1. Norwegian National Advisory Unit on Prehospital Emergency Medicine (NAKOS), Oslo University Hospital, Oslo, Norway; 2. Faculty of Medicine, Institute of Clinical Medicine, University of Oslo, Norway; 3. Department of Health, Medicine and Caring Sciences, Linköping University, Linköping, Sweden; 4. Swedish Civil Contingencies Agency 651 81 Karlstad, Sweden; 5. Norwegian Armed Forces Joint Medical Services, Oslo, Norway

**Keywords:** disaster, guideline, major incident, mass-casualty incident, reporting

## Abstract

**Introduction and Objective:**

Scientific reporting on major incidents, mass-casualty incidents (MCIs), and disasters is challenging and made difficult by the nature of the medical response. Many obstacles might explain why there are few and primarily non-heterogenous published articles available. This study examines the process of scientific reporting through first-hand experiences from authors of published reports. It aims to identify learning points and challenges that are important to address to mitigate and improve scientific reporting after major incidents.

**Methods:**

This was a qualitative study design using semi-structured interviews. Participants were selected based on a comprehensive literature search. Ten researchers, who had published reports on major incidents, MCIs, or disasters from 2013-2018 were included, of both genders, from eight countries on three continents. The researchers reported on large fires, terrorist attacks, shootings, complex road accidents, transportation accidents, and earthquakes.

**Results:**

The interview was themed around initiation, workload, data collection, guidelines/templates, and motivation factors for reporting. The most challenging aspects of the reporting process proved to be a lack of dedicated time, difficulties concerning data collection, and structuring the report. Most researchers had no prior experience in reporting on major incidents. Guidelines and templates were often chosen based on how easily accessible and user-friendly they were.

**Conclusion and Relevance:**

There are few articles presenting first-hand experience from the process of scientific reporting on major incidents, MCIs, and disasters. This study presents motivation factors, challenges during reporting, and factors that affected the researchers’ choice of reporting tools such as guidelines and templates. This study shows that the structural tools available for gathering data and writing scientific reports need to be more widely promoted to improve systematic reporting in Emergency and Disaster Medicine. Through gathering, comparing, and analyzing data, knowledge can be acquired to strengthen and improve responses to future major incidents. This study indicates that transparency and willingness to share information are requisite for forming a successful scientific report.

## Introduction

There is an increasing number of major incidents, mass-casualty incidents (MCIs), and disasters happening all over the world.^1^ The United Nations Sendai Framework for Disaster Risk Reduction 2015-2030 states that countries globally need to increase disaster preparation in order to be better prepared.^2^ Learning from the experience of others is essential, and there is an increasing number of scientific papers published in the field of Disaster Medicine. The challenge is that there is limited available comparable literature reporting from specific incidents, making scientifically grounded decisions for disaster management difficult.

The Sendai Framework plan for disaster management states the importance of science-based preventive means to enhance resilience to disasters both locally and internationally. All stages of disaster management, meaning mitigation, preparedness, response, and recovery, need to have a communicative and uniform method for scientific reporting. However, gathering data from major incidents, MCIs, and disasters is unquestionably difficult.^3^ Case reports have long been the way to learn and share information from these incidents. The heterogenic nature of case reports often makes comparison impossible. Studies have shown that learning from major incidents is challenging even though scientific reports are published in accessible journals.^4^

There has been a call for more systemized and synchronized scientific reporting in literature from researchers, editors, and clinicians.^2–8^ Several available tools for structuring reports on the medical management of the prehospital response have been developed, but few of them have been widely used.^3,4,8–22^ To the authors’ knowledge, there is only one study identifying challenges during the process of reporting through interviewing authors of one specific template.^23^

This study aims to explore and discuss the experience and learning points from the process of scientific reporting on major incidents, MCIs, and disasters.

## Methods

A comprehensive literature search identified available guidelines and templates for structuring scientific reports after major incidents (Appendix A; available online only).^3,4,8–21^ Included in the search were tools for structuring case reports, templates for reporting, and scoring systems for evaluating medical management. The term “guideline” is used for the structuring tools of case reports and case studies, while “template” is used for quantitatively formed structuring and scoring systems.

Consolidated Criteria for Reporting Qualitative Research (COREQ) were developed (Appendix B; available online only). The interview guide (Appendix C; available online only) was developed based on available literature relevant to Emergency Medicine reporting.^3,5,7,8,12,23–28^ Researchers were identified, and a semi-structured interview methodology was chosen to gather new insight and elements around the process of reporting.^28^

### Identification of Researchers

A comprehensive search in Medline (US National Library of Medicine, National Institutes of Health; Bethesda, Maryland USA), PubMed (National Center for Biotechnology Information, National Institutes of Health; Bethesda, Maryland USA), Embase (Elsevier; Amsterdam, Netherlands), and ten scientific reporting databases was conducted for identifying published reports from 2013-2018.^29–38^ Search combinations with the terms “emergency medicine,” “disaster medicine,” “emergency medical service,” “prehospital,” “Pre-hospital,” “reporting,” “case study,” “case report,” “case series,” “major incident,” “mass casualty incident,” and “disaster” (full search string available in Appendix D; available online only) yielded a total of 944 citations, of which 410 were non-duplicates (Table 1).

**Table 1.** Literature Search

1	("2013/01/01"[PDat] : "2017/12/31"[PDat])
2	(Emergency Medical Service*[af] OR Prehospital*[af] OR Pre-hospital*[af])
3	(Emergency Medicine*[af] OR Disaster Medicine*[af])
4	reporting*[af]
5	(case-stud*[af] OR case-report*[af] OR case-serie*[af])
6	(major incident*[af] OR mass casualty incident *[af] OR disaster *[af])
7	1 and 2 and 4 and 6
8	1 and 2 and 5 and 6
9	1 and 3 and 4 and 6
10	1 and 3 and 5 and 6

Note: Full search strings and list of scientific reporting databases in Appendix D (available online only).

**Figure 1.**
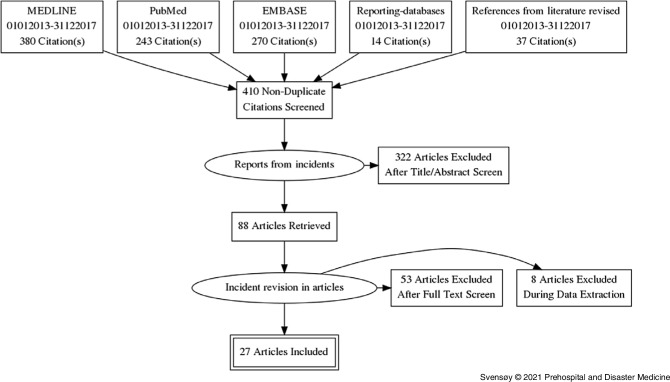
PRISMA Flow Diagram. Abbreviation: PRISMA, Preferred Reporting Items for Systematic Reviews and Meta-Analyses.

Preferred Reporting Items for Systematic Reviews and Meta-Analyses (PRISMA) guidelines were used for the identification of published reports (Figure 1).^39^ From the selected papers, references were investigated. A total of 88 articles were retrieved and screened. Sixty-one were excluded during full-text screen and data extraction as they did not meet inclusion criteria. Several articles were excluded as they did not categorize as a major incident or lacking the prehospital medical aspects. Twenty-seven articles (Figure 1) were included. Authors were identified and contacted for an interview.

### Researchers

The corresponding author from the included papers was contacted. In three of the papers, contact information for the first or corresponding author was not available; in these cases, the last author was contacted. Four articles were excluded as contact information was not obtainable. A total of 23 invitations to participate in this study were sent, one per included article. A total of ten researchers agreed to participate, of which all were interviewed. The researchers included both genders. There were two full-time researchers and eight doctors from a total of eight countries on three continents. The majority of the researchers were directly involved in the health response to the incident they reported. Incidents included large fires, terrorist attacks, shootings, complex road accidents, transportation accidents, and earthquakes—none of the researchers reported on the same incident.

### Ethics Approval and Consent to Participate

All interviewed researchers provided verbal and written consent to participate in this study. Ethical approval was given by the Committee of Medical Ethics at Vrije Universiteit Brussel (VUB; Ixelles, Brussels) February 2018 with individual registration number B.U.N. 143201835043. All data collection is following the guidelines for researchers made by the Commission for the Protection of Privacy (CCP) in Belgium (HM003040430/VT005085301, January 2018).

### Semi-Structured Interviews

The interview guide (Appendix C; available online only) was themed based on available literature on Emergency Medicine reporting.^3,5,7,8,12,23–28^ Major themes included “initiation process,” “workload,” “involvement,” “guidelines/templates,” and “retrospective evaluation.” The interview guide invited the researchers to openly talk about their experience and reflect on obstacles and challenges during their experience on the scientific reporting.^28,40^

Interviews were performed in Norwegian, Swedish, or English. During the thematic analysis approach, quotes translated to English had been back-translated to ensure translation accuracy. Some quotes have been slightly rewritten for anonymization.^41–43^

Personal data were only collected to ensure a heterogeneous population. The participants could add follow-up questions/answers after the interview.

## Results

This study presents findings from the interviews conducted. The main findings were categorized as: (1) motivation the researchers had for reporting; (2) challenges met during the process of reporting; and (3) experience the researchers had with guidelines and templates.

### Motivation

The researchers’ motivation for reporting was often personal, meaning the individual took the initiative rather than the researchers’ employer, management, or government (Table 2). Some were motivated by colleagues or through journals that were promoting scientific reporting. Several researchers were finishing an academic degree and were motivated to publish a paper. Many projects started with the independent initiative from one person or a smaller workgroup rather than an administrative body. One researcher said:

Yes, it was pretty soon afterward - some days afterward - that we found out that we have to write about this. We had one common debrief for the whole prehospital division where we, in the hallway afterward, discussed that we definitely should write something about this.

**Table 2.** Motivation Factors Found Among the Researchers, Not Presented in Hierarchical Order

Motivation Factors for Scientific Reporting:	
	Finishing degree
	Publication
	Increase development in emergency- and disaster medicine
	Wish and feeling of responsibility to share experiences
	Personal engagement in emergency- and disaster medicine
	Motivation through employer or colleagues
	Wanting to tell the story
	Fame

Strong motivation factors were conscience and responsibility towards the medical field, sharing experiences, telling the story of the incident, and forwarding “lessons learned” for developing better responses during major incidents. Many researchers pointed out a wish for writing a useful scientific report, sometimes together with a personal desire for glory or gain. One researcher emphasized:

Is it “one-time glory” or that someone will make use of it? Among us, it was a bit of both.

The motivation for improving and “setting a standard” of reporting outweighed personal goals in most researchers. As expressed by one researcher:

Most of the reports are not uniform, that is why I wanted to do this study.

If you are not willing to share such data and show strengths and weaknesses, then you do not learn anything, then you will not get any better.

### Challenges

Researchers reported that they were not provided with sufficient dedicated time nor funding for the reporting (Table 3). As stated by one researcher:

It was during work, out-of-hours work, spare time, there was no deposited time, it was a completely self-initiated project. Without anything but informal support from the management. Not official in any way.

**Table 3.** Challenges Experienced during Scientific Reporting, Not Presented in Hierarchical Order

Challenges to be Aware of When Reporting on Major Incidents:	
	Time-consuming work
	Getting dedicated time/enough dedicated time
	Funding
	Obtaining allowance to write the report
	Obtaining ethical approval
	Workgroup
	Inconsistent data
	Gather data, especially from other districts and hospitals
	Guideline- or template specific difficulties
	Selection bias

Data collection was particularly tricky, as documentation often was sparse during the response to the incident. Piecing this puzzle together was challenging and time-consuming as data were not always accurate or consistent among informants/systems, often due to the retrospective nature of reporting:

I think that is also a major problem during disaster management research - it is always second-best research. It is second best because it is retrospective. Ours was missing data as well, so it is definitely not optimal.

Obtaining permission and ethical approval for collecting data from their own hospital or district was time-consuming, often delaying the process. Some found that having a larger workgroup and involving the management made it easier to get permission:

We were very thorough to ensure commitment; I think it was important also if you are going to get data and be trusted that the data will not be used in the wrong way, you need to have the management on your side.

Data collection, such as the exact time of the incident, patient data, and the number of ambulances, was expressed as ambiguous, often inaccurate, and inconsistent. The data collection was based on the researchers’ data collected from the incident, data documented in electronic health systems, interviews with personnel/patients/bystanders, press photos, and GPS-data. Qualitative methods were used to gather information after an incident, often through an open invitation to all responders to share their information with the researchers. Collecting data retrospectively through interviews can lead to subjective inaccuracies:

And the other thing is, of course, the data collection, so finding accurate data. If you are interviewing someone retrospectively there will always be inaccuracies, so surely the best way to collect data is in real-time, actually during the incident.

Obtaining permission to use data was easier for researchers associated with the prehospital district or admitting hospital of the incident than for those working in another district or hospital. As described by one researcher:

…as soon as I tried to gather the information from other regions, I just ran into a wall. They would not even respond to my emails. Saying thank you for the email but, we are not going to give you the information.

Skepticism towards the use of the gathered data was the main obstacle for not getting permission to access the data. Regarding interviewing responders for gathering data from an incident, one researcher explained:

I mean some of the interviewees were very cautious; like why do you need this data, can I see it once you have printed it, can you make sure that you do not put this in it. People did question it; because they did not know me; they did not trust me; they did not know what I would write. A lot of the data that they give you is quite incriminating for some people. And I am sure a lot of stuff went wrong that they did not tell me, because it has implications for blame and how things are done.

Language and terminology were one challenge the researchers had with guidelines and templates. Others found some of the guidelines and templates to be time-consuming, not relevant, too comprehensive, or asking for data that was difficult to obtain.

### Guidelines and Templates

The researchers interviewed in this study had experience using six different guidelines or templates. These were guidelines or templates designed for writing reports after major incidents, MCIs, and/or disasters. Only a few researchers had some previous knowledge about the specific guideline or templates they used for reporting (Table 4). The choice of guidelines was often made after the initiation of writing a report. Several of the researchers did not have extensive knowledge about the different reporting tools, so the accessibility of the guideline or template was often a decisive factor. As one researcher stated:

We were pretty inexperienced. It really was what we found; then we at least had something that was recommended.

**Table 4.** Factors Underlying the Choice of Which Guidelines to Use, Not Presented in Hierarchical Order

Choice of Which Guidelines to Use was Based on These Factors:	
	Previous knowledge about the guidelines or templates
	Accessibility
	Amount of work anticipated
	Usability
	Recommendations from colleagues
	Promotion by journals
	Possibility of publication
	Corresponding to the researcher’s motivation

Some decided to use structuring tools promoted by journals, which often promised to ease the publishing process.

Previous knowledge and engagement in the field and the available time and workload anticipated affected how the researchers structured the report. All the researchers wanted more homogenous reporting but emphasized that:

As an individual reporter, one is prone to choose the easiest and most accessible one.

As the research often was performed out of work hours, the required time and complexity of the guidelines or templates may have played a crucial role. Several researchers called for more scientific reports to be published, preferably using a template or guideline to make them comparable.

The structure was often chosen based on the possibility of publishing the report. However, having the possibility to compare and analyze it with other reports was an important aspect emphasized. One researcher said:

I suppose you are looking for short-term gain, short-term benefits as a writer of a report and probably the most prestigious comes from writing case reports. It is a lot more interesting to read; it is more likely to get published straight up, which gives you the immediate reward for writing it. But for filling in a template, that is contributing to a database, for something that is going to be established and accumulate over time, and it may take many years, but it will not give you an immediate reward.

## Discussion

This study presents areas of improvement to the process of scientific reporting based on first-hand experience from researchers who have published articles on specific major incidents, MCIs, and disasters. There are few published articles after major incidents, and there is a considerable discrepancy in the ways of reporting making scientific comparison difficult. This is not to blame the scientific community of Disaster Medicine, as several guidelines and templates are developed in response to heterogeneous scientific reporting (Appendix A; available online only). Although available, the use of these guidelines and templates is sparse.^8,9,22^

Some journals encourage the use of a specific guideline or template for accepting scientific papers for publication.^12^ This practice is not agreed upon among journals; however, it promotes the use of guidelines or templates among reporters. Using the guideline promoted by a journal may provide a more predictable publication process for the author.^5^

As demonstrated by this study, many scientific reports are initiated by individual researchers with great motivation to share knowledge and important lessons learned from the response. Desirably, scientific reporting should be initiated by a department or district rather than an individual. Furthermore, the cooperation between departments and districts eliminates double-reporting by several organizations. Ultimately, every major incident meeting some set criteria should be reported, rather than only those portrayed as big and spectacular.^15^ As a consequence, this could cause a selection bias in that one may not know if a particular incident was left out because things did not work out as planned, were too complex, or because of economic reasons or others. The literature search yielded few reports and often several reports from the same incident. Major incidents that “hit the news” might be more prone for a journal to publish, motivating researchers to select these.

Retrospective data collection in major incidents can be complicated detective work. Patients are often transported to several different hospitals, making patient-specific data collection difficult. Using, or at least having a general knowledge of, a guideline or template while gathering data can mitigate the process by knowing which data to acquire. Getting permission from an organization for collecting data may be facilitated by referring to a specific guideline or template.

As data collection is mostly retrospective, approval for collecting data from the ethics committee should not cause any problems or delays. It is essential to specify the importance of these scientific reports for the global community of Emergency Medicine. Management may mitigate preparation to report through, if possible, getting prospective ethics approval. Data collection from other hospitals than those the researchers in this study were directly involved with was stated to be challenging. Establishing a larger workgroup, with participants spread among the hospitals and districts involved, could facilitate obtaining permission for gathering data. An interdisciplinary workgroup consisting of, for example, doctors, paramedics, nurses, researchers, and management would give more strength to the report, and it would be less prone to professional bias. The various responders to a major incident will have different roles and observe different areas of the incident, which might be equally important in the reporting.

Retrospective data collection from major incidents will often contain inaccuracies, as data often are based on the informants’ memory. In addition, there may be inconsistencies among informants’ descriptions: who was the first responder on the scene or who declared a major incident. Real-time documentation during the response is complicated, but recording devices such as glasses with cameras, voice recorders, and barcode-scanning of patients are being developed.^44^ Collecting data from responders at the scene, or those being a part of the response, can be met with skepticism if the researcher and the informants’ roles are not clearly defined towards how the data will be used and projected. During disasters, there are, by definition, not enough resources and/or personnel. Parts of the response are expected not to go as planned; in other words, the response might not be optimal. Researchers should be protected from harm by sharing data that could be incriminating for others. Some researchers may worry about the implication of telling the truth. Blame, redundancies, or even criminal charges can be the result based on information from scientific reports. Based on this, researchers or informants must not be held accountable for sharing their information.

Another challenge of data collection is the security aspect during or after terrorism or acts of war, where there could be confidential concerns during the incident and investigation afterward. However, based on available papers from major incidents, terrorist attacks, or incidents including an investigation, these concerns do not seem to have any implications on publishing reports; to the authors’ knowledge, rather the opposite.

After a major incident, being involved in the response can be a life-changing event, even for trained personnel.^7,25^ The desire to share one’s story is an essential part of the individual’s debriefing process and coping mechanism. Both the researchers and the informants involved in the response saw contributing to a report as valuable. Not getting the chance to share one’s experience can be demotivating. This underlines that scientific reporting should be a standard part of debriefing, both for the value of information and debriefing itself.

Writing scientific reports is time-consuming work. Dedicated time must be provided to researchers. The choice of which guideline or template to use should not be based on finding the least comprehensive one. However, this is prone to happen if the researchers are not provided with enough dedicated time. More journals should promote the use of guidelines for uniform reporting. Sharing reports through open access publications or a common sharing platform could be valuable in increasing the possibility of comparison and analytical processing.

Case reports have the strength of catching the essence of the incident. A case report may contain crucial information that may be lost when following a template for data gathering. For example, bystander-provided support and anecdotes that complete the whole picture. A case study should cover a minimum of information to be sure the whole picture is not missed. Following a guideline might help the inexperienced and make case studies more comparable.^45^ If one were to write both a case report and fill out a template, researchers interviewed in this study suggested writing a case report to present the whole picture and then using the case report to fill out the template.

This study demonstrates a need for further research on implementing uniform standards for scientific reporting major incidents, and a need to promote reporting to increase the pool of available reports. Open collaboration and information sharing, regionally and globally, are essential for advancing research in Emergency and Disaster Medicine.

## Limitations and Further Research

This study represents a limited study population and presents the view of the individual researcher. Group interviews or questionnaire-type studies, including potential researchers, to further examine the specific elements the researchers presented in this study would be highly valuable.

## Conclusion

After major incidents, writing and publishing scientific reports is the key to sharing knowledge on how to prepare, respond, and learn from other incidents globally. This qualitative study presents experiences from scientific reporting on major incidents, MCIs, and disasters. Through the researchers’ view, this study demonstrates factors that need to be addressed to enhance reporting. The data show that evaluators and researchers need to be given adequate and dedicated time, as well as stakeholders’ support to succeed. There is a consensus in the scientific field that learning outcomes from incidents needs to be studied with a scientific approach to improving future major incident response. Researchers interviewed in this study often personally initiated the process of reporting after responding to an incident, many without support from their organization. Acknowledgment and appreciation for this personal motivation and engagement are essential. Through the already challenging data collection, researchers interviewed call for supportive data sharing from prehospital divisions, hospitals, interdisciplinary rescue organizations, and other responders.

Several structural tools are available for gathering data and writing scientific reports after major incidents, although this study shows that these need to be more widely promoted, especially in the Emergency and Disaster Medicine community. Through gathering, comparing, and analyzing data, knowledge can be acquired to strengthen and improve responses to future major incidents. This study indicates that transparency and willingness to share information and data are requisite for a successful scientific report.

## Supplementary material

For supplementary material accompanying this paper visit https://doi.org/10.1017/S1049023X21000911. click here to view supplementary material
