# Supplementary material for: A Qualitative Study on Researchers’ Experiences after Publishing Scientific Reports on Major Incidents, Mass-Casualty Incidents, and Disasters
Source: Prehosp Disaster Med. 2021 Sep 6;36(5):536–42. doi: 10.1017/S1049023X21000911 (PMC8459171; doi:10.1017/S1049023X21000911)
Supplement: Supplementary file 1 [file S1049023X21000911sup.zip › S1049023X21000911sup001.docx]

| **Utstein-style template** [9] |
| --- |
| Utstein-style template for uniform data reporting of acute medical responses in disaster. |
|  |
| **Majorincidentreporting.net** [10]  Template for reporting of prehospital major incident medical management. |
| **DISAST-CIR** [11] |
| Template for reporting on mass casualty incident in the registry of the Israeli Defence Force Home Front and Ministry of Health. |
|  |
| **Lennquist** [12] |
| Protocol for reports from major accidents and disasters in the international journal of Disaster Medicine. |
|  |
| **Kulling et al**. [8] |
| Guideline for reporting on health crises and critical health events. |
|  |
| **Juffermans, Bierens** [4] |
| Systematic analysis and comparison of the medical response based on eight after-action reports of five consecutive disasters. Identified themes for comparison of reports. |
|  |
| **CONFIDE** [3] |
| Guidelines on reports of field interventions in disasters and emergencies. |
|  |
| **Anderson** [13] |
| Comparative analysis of the emergency medical services and rescue responses to eight airline crashes. |
| **Radestad et al.** [14]  Key indicators for disaster medical response for major incidents. |
| **Castro Delgado et al**. [15] |
| Template used for reporting mass casualty incidents. |
|  |
| **Ricci, Pretto** [16] |
| Assessment of prehospital and hospital response in a disaster. |
| **Daftary et al.** [17]  Formulation of measures of effectiveness for Natural Disaster acute phase medical response. |
| **Performance indicators** [18, 19] |
| Performance indicators for evaluation of major incident medical management. |
|  |
| **Evaluation of disaster and MCI exercises** [20, 21] |
| Systematic evaluation and reporting on exercises |

**APPENDIX A – Guidelines and templates for scientific reporting on Major Incidents, Mass Casualty Incidents, and Disasters.**
