# Supplementary material for: A Qualitative Study on Researchers’ Experiences after Publishing Scientific Reports on Major Incidents, Mass-Casualty Incidents, and Disasters
Source: Prehosp Disaster Med. 2021 Sep 6;36(5):536–42. doi: 10.1017/S1049023X21000911 (PMC8459171; doi:10.1017/S1049023X21000911)
Supplement: Supplementary file 1 [file S1049023X21000911sup.zip › S1049023X21000911sup003.docx]

**APPENDIX C – Interview Guide**

**A qualitative study on researchers' experiences after publishing scientific reports on Major Incidents, Mass Casualty Incidents, and Disasters.**

Interview guide

Duration: 35-60 minutes (introduction/personal data/reporting experience 5-10 min, interview 30-40 min, end points/additional points 0-10 min)

Initiation process

- Open question about the reporting work done after the incident
- When
- Initiative
- Workgroup
- Timeframe

Involvement

- Workload
- How much time was used?
  - Freetime
  - Work
- Division of work
- Data collection
- Ethics
- Approval

Guidelines/Templates

- Previous knowledge about guidelines or templates, from where
- Using guidelines or templates
  - Positives/negatives
  - Quantitative/Qualitative
- Motivation for using/not using

After reporting

- Motivation for reporting again – from yourself and/or others
- Would you do something differently next time
- Feedback
- Changes (system/local) based on report/lessons learned

Endpoints
