# Supplementary material for: A Qualitative Study on Researchers’ Experiences after Publishing Scientific Reports on Major Incidents, Mass-Casualty Incidents, and Disasters
Source: Prehosp Disaster Med. 2021 Sep 6;36(5):536–42. doi: 10.1017/S1049023X21000911 (PMC8459171; doi:10.1017/S1049023X21000911)
Supplement: Supplementary file 1 [file S1049023X21000911sup.zip › S1049023X21000911sup004.docx]

| 1 | ("2013/01/01"[PDat] : "2017/12/31"[PDat]) |
| --- | --- |
| 2 | (Emergency Medical Service*[af] OR Prehospital*[af] OR Pre-hospital*[af]) |
| 3 | (Emergency Medicine*[af] OR Disaster Medicine*[af]) |
| 4 | reporting*[af] |
| 5 | (case-stud*[af] OR case-report*[af] OR case-serie*[af]) |
| 6 | (major incident*[af] OR mass casualty incident *[af] OR disaster *[af]) |
| **7** | 1 and 2 and 4 and 6 |
| **8** | 1 and 2 and 5 and 6 |
| **9** | 1 and 3 and 4 and 6 |
| **10** | 1 and 3 and 5 and 6 |

**APPENDIX D – Search string**

| Search strings: |
| --- |
| **7** = “((Emergency Medical Service* or Prehospital or Pre-hospital) and reporting and (major incident* or mass casualty incident* or disaster*))” |
| **8** = “((Emergency Medical Service* or Prehospital or Pre-hospital) and (case-stud* OR case-report* OR case-serie*) and (major incident* or mass casualty incident* or disaster*))” |
| **9** = “((Emergency Medicine* OR Disaster Medicine*) and reporting and (major incident* or mass casualty incident* or disaster*))” |
| **10** = “((Emergency Medicine* OR Disaster Medicine*) and (case-stud* OR case-report* OR case-serie*) and (major incident* or mass casualty incident* or disaster*))” |

In addition to PubMed, ten reporting databases were included in the search; Emergency Events Database (EM-DAT) [29], The Major Accident Reporting System (eMARS) [30], ECCAIRS - European Co-ordination Centre for Accident and Incident Reporting Systems [31], The Accident Investigation Board Norway (Norway) [32], Majorincidentreporting [33], Kamedo-reports (Sweden) [34], Failure and Accidents Technical information System (Netherlands) [35], Ship/Platform Collision Incident Database (UK HSE report) [36], The National Transportation Safety Board (NTSB) [37], and European Union Railway accident and incident investigations [38]. From these databases, only scientific reports focusing on the medical aspect were included.
